# EXPLORING THE EFFICACY OF MOCA SCORE CORRECTIONS IN REDUCING THE INFLUENCE OF RACE/ETHNICITY

**DOI:** 10.1093/geroni/igac059.2477

**Published:** 2022-12-20

**Authors:** Rachel McCray, Omonigho Bubu, Judite Blanc, Azizi Seixas, Girardin Jean-Louis, Arlener Turner

**Affiliations:** New York University, New York City, New York, United States; NYU Grossman School of Medicine, New York, New York, United States; University of Miami, Miami, Florida, United States; University of Miami, Miami, Florida, United States; University of Miami, Miami, Florida, United States; University of Miami, Miami, Florida, United States

## Abstract

As studies have highlighted significant differences in test score distributions between ethnicities, we chose to examine if the MoCA corrections for education curb racial differences. Therefore, we use data from the NIA Alzheimer's Disease Research Center (ADRC) program to explore the efficacy of score corrections in reducing the influence of race/ethnicity. This study utilized the NACC dataset to analyze data covering UDS visits from September 2005 to February 2021. Participants included in the analyses (n= 11987, 64.9% women, 12.3 % Black/African American, mean age 73□9.460; 16□4.98 years of education) were all cognitively normal. The analyses uses the Montreal Cognitive Assessment (MoCA) with and without correction for education (addition of one point for less than 12 years of education), via cut off score derived cognitive status categories. A 2x3 contingency table revealed a statistically significant association between participants’ race (black vs white) and performance on the uncorrected MoCA, X22,n=5291=188.971, p<.001, and the corrected MoCA X22,n=5282=167.073, p<.001. Additionally, a One-way ANCOVA analysis comparing the correlation of education and uncorrected MoCA score for Black/African American (r=.425, p<.001) and White participants (r=.198, p<.001) shows a significant difference between the two groups F1,5288=167.992, p<.001. Specifically, in Black/African American participants, the correlation is much stronger suggesting that years of education is a greater determinant of cognitive status. These results demonstrate that regardless of controlling for education via adding buffer points significant racial disparities in global cognition scores were still present. Alternative corrections for race and education should be considered for future test adaptations.

